# Association of progesterone production with serum anti-Müllerian hormone levels in assisted reproductive technology cycles with corifollitropin alfa

**DOI:** 10.1371/journal.pone.0206111

**Published:** 2018-11-14

**Authors:** Tsung-Hsien Lee, Shu-Ling Tzeng, Chun-I Lee, Hsiu-Hui Chen, Chun-Chia Huang, Shee-Uan Chen, Maw-Sheng Lee

**Affiliations:** 1 Institute of Medicine, Chung Shan Medical University, Taichung, Taiwan; 2 Department of Obstetrics and Gynecology, Chung Shan Medical University Hospital, Taichung, Taiwan; 3 Department of Obstetrics and Gynecology, National Taiwan University and National Taiwan University Hospital, Taipei, Taiwan; 4 Division of Infertility Clinic, Lee Women’s Hospital, Taichung, Taiwan; University of Mississippi Medical Center, UNITED STATES

## Abstract

The use of corifollitropin alfa (CA) in assisted reproductive technology (ART) cycles is dependent on the antral follicle count and body weight of patients. The present study investigated the safety and efficacy of using 100μg of CA in predicted excessive responders based on serum anti-Mullerian hormone (AMH) level. The results of 381 ART cycles stimulated by CA versus daily recombinant follicle-stimulation hormone (rFSH) in patients with low (<1.0 ng/mL; n = 38 vs. n = 90), moderate (1.0–3.36 ng/mL; n = 38 vs. n = 95), and high (> 3.36 ng/mL; n = 48 vs. n = 72) serum AMH levels, were analyzed. Pregnancy and live birth rates did not significantly differ between CA and daily rFSH groups. In the patients with high AMH levels, serum progesterone (P4) levels on the day of human chorionic gonadotropin (hCG) injection were significantly lower in the CA group than in the rFSH group (0.93 ± 0.55 vs. 1.16 ± 0.64 ng/mL). Furthermore, serum P4 levels on the day of hCG injection were negatively correlated with baseline AMH levels in the CA group, but not in the rFSH group, in the patients with high AMH levels. In conclusion, the use of 100 μg of CA in patients with high AMH levels is safe and effective and is associated with a lower P4 level on the day of hCG injection compared with the use of daily rFSH.

## Introduction

Corifollitropin alfa (CA), a novel recombinant molecule exhibiting long-acting follicle stimulating hormone (FSH) bioactivity, was recently developed for clinical use in assisted reproductive technology (ART) cycles. One injection of CA can replace seven injections of daily recombinant FSH (rFSH) used for controlled ovarian stimulation in ART cycles [1]. Several randomized clinical trials including Engage [2], Ensure [3], and Pursue [4] have compared the safety and efficacy of CA with those of daily rFSH in a gonadotropin releasing hormone (GnRH) antagonist protocol used in ART cycles for normal or poor responders.

The use of CA is based on the antral follicle count (AFC) and body weight of patients. The use of CA is recommended only in patients with an AFC of <20 according to manufacturer’s instructions. The choice of 100 or 150 μg of CA depends on the body weight (<60 or >60 kg, respectively) of patients [5]. Although some clinical modifications in ART treatment were performed in the Engage trial without affecting the pregnancy outcome [6], information regarding predicted excessive responders is lacking.

A meta-analysis reported that the risk of ovarian hyperstimulation syndrome (OHSS) was higher in the CA protocol than in the daily rFSH protocol [7]. However, similar risk of OHSS has been reported in subsequent meta-analyses [8, 9]. Here we propose that the use of 100 μg of CA and the subsequent administration of a low dose of rFSH can be a patient friendly protocol for predicted excessive responders in ART cycles and would not increase the risk of OHSS.

Both anti-Müllerian hormone (AMH) and the AFC are strong predictors of the ovarian response of patients undergoing ART cycles with the use of daily rFSH [10–13]. In our previous study, we found that an AMH level of >3.36 ng/mL was associated with an increased risk of OHSS [14]. In addition, AMH has been shown to be beneficial in predicting a low or excessive response when using CA in GnRH antagonist protocols [15, 16]. These results suggest that AMH can be used to predict the ovarian response and consequently to select the CA dose in a GnRH antagonist protocol for AMH-predicted excessive responders.

An AMH stratification for tailoring the gonadotropin dose was developed for application to a long GnRH agonist protocol and a GnRH antagonist protocol involving daily rFSH administration [17]. Furthermore, the AMH approach was used in a randomized controlled study of a new rFSH [18]. However, the AMH approach for the use of CA has rarely been investigated.

The present study investigated the safety and efficacy of applying the AMH approach to guide the use of CA in a GnRH antagonist protocol, particularly for AMH-predicted excessive responders (AMH levels > 3.36 ng/mL) with the use of 100μg of CA. In addition, differences in follicular development and steroid hormone profiles on the day of human chorionic gonadotropin (hCG) administration between CA and daily rFSH treatments were examined.

## Materials and methods

We conducted this case-control study to investigate the relationship between AMH and the ovarian responses and pregnancy outcomes of ART cycles with CA treatment. The study protocol was approved by the Institutional Ethics Review Board of Chung Shan Medical University Hospital (CS12248 and CS2-14033). Patients with infertility were enrolled after a standard medical workup to determine whether treatment with in vitro fertilization (IVF) or intracytoplasmic sperm injection (ICSI) was required. Written informed consent for study participation was obtained from the patients. The participants were assigned to three treatment doses according to their serum AMH levels prior to stimulation cycles. We excluded women who had uterine factor infertility or ovarian cysts, as determined through ultrasound and hysteroscopy prior to the commencement of a stimulation cycle.

**Fig 1:**
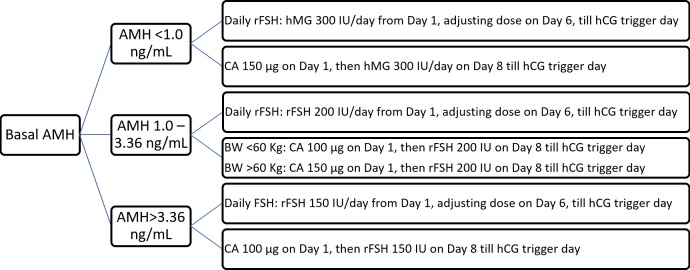
Protocols of corifollitropin alfa (CA) versus daily gonadotropin [human menopausal gonadotropin (hMG) or recombinant follicle-stimulating hormone (rFSH)] with the use of GnRH antagonist through anti-Müllerian hormone (AMH) stratification. The GnRH antagonist was administered on sixth day of stimulation till the day of human chorionic gonadotropin (hCG) injection.

Subsequent to counseling for ART treatment, we selected the GnRH antagonist protocol with two types of initiated gonadotropin formula, either CA or conventional rFSH. Patients who were administered CA were considered as the case group, and those who were administered daily rFSH were considered as the control group. The initial dose of CA or rFSH was determined according to the patients’ basal AMH levels (Fig 1).

### Protocols for patients with low AMH levels (AMH < 1.0 ng/mL)

The GnRH antagonist (cetrorelix) protocol was performed as described previously [19] with minor modification. The stimulation process was started with the administration of 300 IU of human menopausal gonadotropin (hMG, Menopur; Ferring, Saint-Prex, Switzerland) every day for 5 days. Subsequently, the hMG dose was adjusted according to the ovarian response. In patients who received long-acting FSH, 150 μg of CA (Elonva; MSD, New Jersey, USA) was administered irrespective of their body weight. The GnRH antagonist (0.25 mg of cetrorelix; Cetrotide; Merk Serono, Anbunne, Switzerland) was administered on the sixth day of hMG stimulation. The ovarian response to this therapy was monitored by measuring serial serum estradiol (E2) levels and through transvaginal ultrasonography. We stopped rFSH administration after observing two leading follicles (at least 17 mm in diameter) and then administered 250 μg of hCG (Ovidrel; Merk Serono).

### Protocols for patients with moderate AMH levels (1.0 ng/mL < AMH < 3.36 ng/mL)

The stimulation protocol was performed as described previously for patients with low AMH levels, except that the daily rFSH group was administered at 200 IU/day of rFSH (Puregon; MSD). We administered 100 and 150 μg of CA in women with a body weight of <60 and >60 kg, respectively. The methodology for monitoring ovarian response and criterion for hCG administration were the same as those used in the protocol for patients with low AMH levels. After an injection of CA, we injected 200 IU of rFSH daily until the day of hCG administration.

### Protocols for patients with high AMH levels (AMH > 3.36 ng/mL)

The stimulation protocol was performed as described previously for patients with low AMH levels, except that the daily rFSH group was administered 150 IU/day of rFSH. We administered 100 μg of CA to all patients in this group irrespective of their body weight. The methodology for monitoring the ovarian response and criterion for the hCG administration were the same as those used in the protocol for patients with low AMH levels. After the injection of CA, 150 IU of rFSH was injected daily until the day of hCG administration. If the serum E2 level was >3,600 pg/mL or >20 follicles (>10 mm in diameter) were observed on the day of hCG trigger, only 3,000 IU of hCG was injected for final oocyte maturation.

### Hormone measurement

The first blood sampling for measuring basal AMH, FSH, LH, and E2 levels was performed on day 2 to3 of the menstruation cycles prior to ovarian hyperstimulation. The second blood sampling for measuring FSH, LH, and E2 levels was performed on day 2 to3 of the stimulation cycle prior to gonadotropin or CA injection. The third blood sampling for measuring E2, LH, and progesterone (P4) levels was performed on the day of hCG administration.

Hormone levels were measured using the same method as that used in our previous study [20]. Briefly, the serum AMH level was measured in duplicate by using the AMH/MIS ELISA kit (Immunotech/Beckman Coulter Inc., Marseille Cedex, France).

Serum FSH, LH, E2, and P4 levels were measured using a specific immunometric assay kit (Access; Beckman Coulter Inc., Fullerton, CA, USA). The minimal detection limit was 20 pg/mL (73 pmol/L) for E2 and 0.08 ng/mL (0.25 nmol/L) for P4.

### Statistical analysis

Various biological parameters related to ART cycles were compared between CA and daily rFSH treatments by using Student’s t test. The rates of fertilization, ongoing pregnancy and live birth were compared using the χ^2^ test or Fisher’s exact test, as applicable.

Multivariable linear regression was performed to determine the independent effect of individual variables, namely age, basal AMH, FSH, LH levels, follicle number, and E2 levels on the day of hCG injection, on P4 levels. All analyses were performed using the Statistical Package for Social Sciences (Version 17.0; SPSS, Chicago, IL). A confidence level of P < .05 was considered significant for comparison purposes.

## Results

A total of 381 couples were recruited from December 2014 to May 2015. These participants were divided into the following three groups according to their AMH levels prior to ovarian stimulation: low (AMH < 1.0 ng/mL; n = 128), moderate (AMH = 1.0–3.36 ng/mL; n = 133), and high (AMH > 3.36 ng/mL; n = 120) AMH groups. The baseline data of the patients with low (n = 38 vs. n = 90), moderate (n = 38 vs. n = 95), and high (n = 48 vs. n = 72) AMH levels who were using CA versus daily rFSH in a GnRH antagonist protocol, respectively, are listed in Table 1. The age, infertility duration, and baseline hormone profiles (FSH, LH, E2, and AMH) did not significantly differ between the CA and daily rFSH groups in the patients with low, moderate, and high AMH levels.

**Table 1:** Baseline data of patients who were using corifollitropin alfa versus daily recombinant follicle-stimulating hormone (rFSH) and were stratified by low (<1.0 ng/mL), moderate (1.0–3.36 ng/mL), and high (> 3.36 ng/mL) AMH levels.

AMH levels (ng/mL)	Low (<1.0)		Moderate (1.0–3.36)		High (>3.36)	
Treatments	Daily rFSH	Corifollitropin alfa	Daily rFSH	Corifollitropin alfa	Daily rFSH	Corifollitropin alfa
**N (cycles)**	90	38	95	38	72	48
**Age (years)**	40.1±3.5	39.9±4.1	38.6±4.1	38.1±3.4	35.0±5.1	33.7±4.4
**Duration of infertility (years)**	4.3±3.4	4.3±3.4	3.4±2.8	3.5±2.9	3.3±3.0	3.0±1.9
**Body Weight (Kg)**	56.5±8.7	56.6±8.4	55.0±7.9	56.8±7.5	55.3±8.3	54.9±6.7
**BMI (Kg/M**^**2**^**)**	22.1±2.9	21.7±2.8	21.6±2.9	22.1±3.0	21.9±2.9	21.3±2.7
**AMH (ng/ml)**	0.53±0.25	0.44±0.26	2.01±0.68	2.19±0.60	5.99±2.52	6.41±2.87
**FSH (IU/L)**	9.6±4.8	8.9±3.8	7.5±3.5	7.3±2.5	6.4±2.4	6.1±2.1
**LH (IU/L)**	5.9±4.3	4.6±3.3	4.9±3.7	5.1±3.1	5.6±3.7	5.1±3.7
**E2 (pg/ml)**	39.7±21.5	37.0±22.2	30.0±20.8	36.4±20.2	29.3±17.3	32.8±19.4

Table 2 shows the stimulation results of the CA versus daily rFSH protocol. In the patients with high AMH levels, serum P4 level (0.93 ± 0.55 vs. 1.16 ± 0.64 ng/mL; P = .044) on the day of hCG administration were significantly lower in the CA group (n = 48) than in the daily FSH group (n = 72). However, the incidence of OHSS, number of retrieved oocytes, pregnancy outcome, and live birth rate did not significantly differ between the CA and daily rFSH groups in the patients with low, moderate, and high AMH levels.

**Table 2:** Outcome of ART cycles with the use of corifollitropin alfa and daily recombinant FSH in patients stratified by low (<1.0 ng/mL), moderate (1.0–3.36 ng /mL), and high (> 3.36 ng/mL) AMH levels.

AMH levels (ng/mL)	Low (<1.0)		Moderate (1.0–3.36)		High (>3.36)	
Treatments	Daily rFSH	Corifollitropin alfa	Daily rFSH	Corifollitropin alfa	Daily rFSH	Corifollitropin alfa
**N (cycles)**	90	38	95	38	72	48
**E2 on the day of hCG injection** **(pg/ml)**	592±497	644±574	1371±1002	1286±786	2876±1836	2378±1187
**P4 on the day of hCG injection** **(ng/ml)**	0.68±0.39	0.67±0.42	0.85±0.45	0.72±0.38	1.16±0.64^a^	0.93±0.55^a^
**Follicle no.**	3.7±2.5	4.1±3.2	8.4±5.1	8.9±3.2	19.2±8.5	16.8±5.6
**Oocyte no.**	2.9±2.6	2.9±3.4	6.8±4.7	8.3±4.0	16.0±6.6	16.0±6.1
**Fertilization rate (%)**	74.9±32.9	66.6±39.3	72.3±28.8	71.6±27.1	74.1±19.3	74.6±16.0
**Zygote no.**	2.1±1.6	2.4±2.6	3.9±3.1	4.5±2.6	10.5±6.5	9.9±5.0
**Good embryo rate (Day2, %)**	61.9±41.0	75.3±33.9	65.9±34.1	60.9±27.0	58.7±25.3	53.2±33.1
**OHSS rate**	0	0	0	0	2.8 (2/72)	2.0 (1/49)
**Embryo transfer no.**	2.1±1.1	2.0±1.0	1.9±0.6	2.1±0.5	1.8±0.5	1.7±0.4
**Ongoing pregnancy rate**	11.1 (10/90)	10.5 (4/38)	28.4 (27/95)	34.2 (13/38)	44.4 (32/72)	54.2 (26/48)
**Live birth rate**	8.9 (8/90)	7.9 (3/38)	22.1 (21/95)	28.9 (11/38)	34.7 (25/72)	45.8 (22/48)

^a^ P = .044 by Student’s *t* test

OHSS denotes ovarian hyperstimulation syndrome.

Linear regression models were used to analyze the association between the baseline serum AMH level and steroid hormone production after controlling ovarian hyperstimulation. The results of the multivariable linear regression analysis (enter model) of P4 levels on the day of hCG administration showed that only serum E2 levels on the day of HCG injection positively correlated with P4 levels in the daily rFSH treatment group (Table 3). By contrast, we observed that serum P4 levels on the day of hCG trigger correlated not only positively with E2 levels on the day of hCG injection but also negatively with basal AMH levels in the CA treatment group for the patients with high AMH levels (Table 3).

**Table 3:** Correlation between the progesterone level on the day of human chorionic gonadotropin (hCG) injection and other baseline characteristics in ART cycles by multivariable linear regression analysis (enter model).

AMH levels	Low (<1.0)		Moderate (1.0–3.36)		High (>3.36)	
Regression coefficient (95% CI)	Daily rFSH	Corifollitropin alfa	Daily rFSH	Corifollitropin alfa	Daily rFSH	Corifollitropin alfa
**AMH (ng/ml)**	-0.171 (-0.483–0.141)	0.003 (-0.554–0.560)	0.142 (0.025–0.259)^a^	-0.113 (-0.303–0.077)	-0.009 (-0.063–0.044)	-0.074 (-0.030- -0.118)^b^
**E2 on hCG day** **(1000pg/ml)**	0.318 (0.159–0.478)^c^	0.266 (0.015–0.518)^a^	0.227 (0.148–0.350) ^c^	0.269 (0.125–0.413) ^b^	0.211 (0.138–0.285) ^c^	0.292 (0.186–0.398) ^c^

^a^ P < .05

^b^ P < .01, and

^c^ P <0.001 by linear regression analysis

## Discussion

The results of the present study suggested that the serum AMH levels affected follicular development and P4 production in the patients with high basal AMH levels receiving CA treatment. In those patients, high baseline AMH levels were associated with low P4 levels on the day of hCG injection in the CA but not in the daily rFSH protocol.

Our study results suggest that 150 μg of CA can be used in patients with low AMH levels regardless of their body weight. The present results are consistent with those reported by Polyzos et al. in 2013 [21]. The dose of CA for patients with moderate AMH levels should be set according to manufacturer’s instructions. Moreover, 100 μg of CA and the subsequent low dose of rFSH may be suitable for patients with high AMH levels without increasing the risk of OHSS or reducing the chance of ongoing pregnancy.

In the predicted excessive responders (AMH > 3.36 ng/mL), P4 levels on the day of hCG administration were significantly lower in the CA group than in the daily rFSH group. These results are similar to those of a study that reported that in patients with excessive responses (>18 oocytes), P4 levels were lower in a CA group than in a daily rFSH group [22]. These findings suggest that follicular development and ovarian steroid hormone production can differ between CA and daily rFSH stimulation for patients with high AMH levels or a high oocyte yield.

The results of multivariable linear regression analysis indicated that high AMH levels were associated with low P4 levels after adjusting with the E2 levels in the CA group but not in the daily rFSH group for the patients with high AMH levels. A pharmacodynamic study of CA reported a rapid rise and a gradual fall of FSH activity after one injection of CA [1]. The dynamic change in FSH activity is similar to the “stepdown” of daily rFSH administration, and granulosa cells in small or poor-quality follicles might undergo apoptosis or atresia in such a condition. Consequently, the number of follicles producing P4 might have been lower in the CA group than in the daily rFSH group. Nonetheless, such a phenomenon was evident only in the group with the highest AMH level.

A study on daily rFSH treatment with a long GnRH agonist protocol reported a negative correlation between the baseline AMH levels and the follicular output rate (the ratio of follicles on the day of hCG injection to small antral follicles on day 3) [23], confirming the hypothesis that AMH inhibits sensitivity to the exogenous rFSH stimulation of ovarian follicles [23]. With a further increase in the AMH levels, the inhibition of FSH activity becomes more prominent and may cause anovulation in women, especially in those with polycystic ovary syndrome (PCOS) [24]. One injection of CA provides adequate FSH bioactivity to overcome the inhibition induced by a high AMH level, and the subsequent stepdown of FSH bioactivity enables selective development among the cohort of follicles. Therefore, P4 production in the CA group was negatively correlated with basal AMH levels.

The risk of OHSS was reported to be higher in patients with high AFC or AMH levels in both CA [25] and daily rFSH groups receiving a GnRH antagonist protocol. In this prospective study, only one participant receiving CA treatment (1/48; 2.1%) and two participants receiving daily rFSH treatment (2/72; 2.8%) developed OHSS in the high AMH group. The incidence of OHSS in this study is similar to that (6–9%) reported in a recent meta-analysis of a GnRH antagonist protocol [26]. Furthermore, the CA protocol may be combined with GnRH agonist trigger and the freeze all policy for patients with PCOS to completely prevent OHSS [27].

A high response in daily rFSH or CA treatment with the use of a GnRH antagonist did not reduce the chance of pregnancy [22]. By contrast, a high P4 level on the day of hCG injection has been associated with a low pregnancy rate [28–31]. We propose that the use of 100 μg of CA in patients with high AMH levels may be associated with a higher live birth rate because of the presence of a low P4 level on the day of hCG trigger.

The limitation of the present study is its pilot scale with a small sample size and a non-randomized design. According to the present results for live birth rates in the patients with AMH levels of > 3.36 ng/mL (45.8% vs 34.7%, Table 2), a randomized clinical trial should be conducted with a larger sample size (n = 306 for CA treatment and n = 306 for daily rFSH group) of patients with high AMH levels to prove the beneficial effects of CA treatment.

In recent years, freeze-all policy has been frequently used for excessive responders to prevent OHSS after the report of a randomized clinical trial conducted in patients with PCOS [32]. We could not collect adequate number of cycles performed with fresh embryo transfer to prove this concept. Nonetheless, the results of the present study may be applicable to patients requiring fresh embryo transfer due to limitation of time, patients with extremely high AMH levels and inadequate responses to 150–300 IU of daily rFSH, and patients with PCOS patients receiving GnRH agonist trigger [27].

In conclusion, the baseline AMH levels may affect follicular development and P4 production in ART cycles using CA in patients with an AMH level of > 3.36 ng/mL. For such AMH-predicted excessive responders, the use of 100 μg of CA is associated with a lower P4 level compared with the use of daily rFSH in ART cycles. Theoretically, such low P4 levels may be beneficial for endometrial receptivity, pregnancy, and live birth rates in patients with high AMH levels. The risk of OHSS is comparable between daily rFSH and CA treatment for such AMH- predicted excessive responders.

## Supporting information

S1 Dataset The anonymized data in the present study. (XLSX)

## References

[1] Fauser BC, Alper MM, Ledger W, Schoolcraft WB, Zandvliet A, Mannaerts BM, et al Pharmacokinetics and follicular dynamics of corifollitropin alfa versus recombinant FSH during ovarian stimulation for IVF. Reprod Biomed Online. 2010;21(5):593–601. 10.1016/j.rbmo.2010.06.032 . 20843746

[2] Devroey P, Boostanfar R, Koper NP, Mannaerts BM, Ijzerman-Boon PC, Fauser BC, et al A double-blind, non-inferiority RCT comparing corifollitropin alfa and recombinant FSH during the first seven days of ovarian stimulation using a GnRH antagonist protocol. Hum Reprod. 2009;24(12):3063–72. 10.1093/humrep/dep291 ; PubMed Central PMCID: PMCPMC2777786. 19684043 PMC2777786

[3] Corifollitropin alfa Ensure Study G. Corifollitropin alfa for ovarian stimulation in IVF: a randomized trial in lower-body-weight women. Reprod Biomed Online. 2010;21(1):66–76. 10.1016/j.rbmo.2010.03.019 . 20483664

[4] Boostanfar R, Shapiro B, Levy M, Rosenwaks Z, Witjes H, Stegmann BJ, et al Large, comparative, randomized double-blind trial confirming noninferiority of pregnancy rates for corifollitropin alfa compared with recombinant follicle-stimulating hormone in a gonadotropin-releasing hormone antagonist controlled ovarian stimulation protocol in older patients undergoing in vitro fertilization. Fertil Steril. 2015;104(1):94–103 e1. 10.1016/j.fertnstert.2015.04.018 . 26003273

[5] de Greef R, Zandvliet AS, de Haan AF, Ijzerman-Boon PC, Marintcheva-Petrova M, Mannaerts BM. Dose selection of corifollitropin alfa by modeling and simulation in controlled ovarian stimulation. Clin Pharmacol Ther. 2010;88(1):79–87. 10.1038/clpt.2010.54 . 20520605

[6] Leader A, Devroey P, Witjes H, Gordon K. Corifollitropin alfa or rFSH treatment flexibility options for controlled ovarian stimulation: a post hoc analysis of the Engage trial. Reprod Biol Endocrinol. 2013;11:52 10.1186/1477-7827-11-52 ; PubMed Central PMCID: PMCPMC3691650. 23758821 PMC3691650

[7] Mahmoud Youssef MA, van Wely M, Aboulfoutouh I, El-Khyat W, van der Veen F, Al-Inany H. Is there a place for corifollitropin alfa in IVF/ICSI cycles? A systematic review and meta-analysis. Fertil Steril. 2012;97(4):876–85. 10.1016/j.fertnstert.2012.01.092 . 22277766

[8] Griesinger G, Boostanfar R, Gordon K, Gates D, McCrary Sisk C, Stegmann BJ. Corifollitropin alfa versus recombinant follicle-stimulating hormone: an individual patient data meta-analysis. Reprod Biomed Online. 2016;33(1):56–60. 10.1016/j.rbmo.2016.04.005 . 27178762

[9] Pouwer AW, Farquhar C, Kremer JA. Long-acting FSH versus daily FSH for women undergoing assisted reproduction. Cochrane Database Syst Rev. 2015;(7):CD009577 10.1002/14651858.CD009577.pub3 . 26171903 PMC10415736

[10] Broekmans FJ, Kwee J, Hendriks DJ, Mol BW, Lambalk CB. A systematic review of tests predicting ovarian reserve and IVF outcome. Hum Reprod Update. 2006;12(6):685–718. 10.1093/humupd/dml034 . 16891297

[11] Broer SL, Dolleman M, van Disseldorp J, Broeze KA, Opmeer BC, Bossuyt PM, et al Prediction of an excessive response in in vitro fertilization from patient characteristics and ovarian reserve tests and comparison in subgroups: an individual patient data meta-analysis. Fertil Steril. 2013;100(2):420–9 e7. 10.1016/j.fertnstert.2013.04.024 . 23721718

[12] Broer SL, Mol BW, Hendriks D, Broekmans FJ. The role of antimullerian hormone in prediction of outcome after IVF: comparison with the antral follicle count. Fertil Steril. 2009;91(3):705–14. 10.1016/j.fertnstert.2007.12.013 . 18321493

[13] Nelson SM, Klein BM, Arce JC. Comparison of antimullerian hormone levels and antral follicle count as predictor of ovarian response to controlled ovarian stimulation in good-prognosis patients at individual fertility clinics in two multicenter trials. Fertil Steril. 2015;103(4):923–30 e1. 10.1016/j.fertnstert.2014.12.114 . 25624196

[14] Lee TH, Liu CH, Huang CC, Wu YL, Shih YT, Ho HN, et al Serum anti-Mullerian hormone and estradiol levels as predictors of ovarian hyperstimulation syndrome in assisted reproduction technology cycles. Hum Reprod. 2008;23(1):160–7. Epub 2007/11/15. 10.1093/humrep/dem254 . 18000172

[15] Oehninger S, Nelson SM, Verweij P, Stegmann BJ. Predictive factors for ovarian response in a corifollitropin alfa/GnRH antagonist protocol for controlled ovarian stimulation in IVF/ICSI cycles. Reprod Biol Endocrinol. 2015;13:117 10.1186/s12958-015-0113-1 ; PubMed Central PMCID: PMCPMC4628292. 26520396 PMC4628292

[16] Polyzos NP, Tournaye H, Guzman L, Camus M, Nelson SM. Predictors of ovarian response in women treated with corifollitropin alfa for in vitro fertilization/intracytoplasmic sperm injection. Fertil Steril. 2013;100(2):430–7. 10.1016/j.fertnstert.2013.04.029 . 23668992

[17] Nelson SM, Yates RW, Lyall H, Jamieson M, Traynor I, Gaudoin M, et al Anti-Mullerian hormone-based approach to controlled ovarian stimulation for assisted conception. Hum Reprod. 2009;24(4):867–75. 10.1093/humrep/den480 . 19136673

[18] Arce JC, Andersen AN, Fernandez-Sanchez M, Visnova H, Bosch E, Garcia-Velasco JA, et al Ovarian response to recombinant human follicle-stimulating hormone: a randomized, antimullerian hormone-stratified, dose-response trial in women undergoing in vitro fertilization/intracytoplasmic sperm injection. Fertil Steril. 2014;102(6):1633–40 e5. 10.1016/j.fertnstert.2014.08.013 . 25256937

[19] Lee TH, Wu MY, Chen HF, Chen MJ, Ho HN, Yang YS. Ovarian response and follicular development for single-dose and multiple-dose protocols for gonadotropin-releasing hormone antagonist administration. Fertil Steril. 2005;83(6):1700–7. Epub 2005/06/14. 10.1016/j.fertnstert.2004.12.037 . 15950639

[20] Lee MS, Tzeng SL, Yang SF, Lin YP, Cheng EH, Huang CC, et al Correlation of serum anti-Mullerian hormone to follicular follicle stimulating hormone and implantation potential of the ensuing embryos. Clin Chim Acta. 2017;471:327–33. Epub 2017/07/08. 10.1016/j.cca.2017.06.023 . 28684220

[21] Polyzos NP, De Vos M, Corona R, Vloeberghs V, Ortega-Hrepich C, Stoop D, et al Addition of highly purified HMG after corifollitropin alfa in antagonist-treated poor ovarian responders: a pilot study. Hum Reprod. 2013;28(5):1254–60. 10.1093/humrep/det045 . 23442756

[22] Fatemi HM, Doody K, Griesinger G, Witjes H, Mannaerts B. High ovarian response does not jeopardize ongoing pregnancy rates and increases cumulative pregnancy rates in a GnRH-antagonist protocol. Hum Reprod. 2013;28(2):442–52. 10.1093/humrep/des389 . 23136144

[23] Genro VK, Grynberg M, Scheffer JB, Roux I, Frydman R, Fanchin R. Serum anti-Mullerian hormone levels are negatively related to Follicular Output RaTe (FORT) in normo-cycling women undergoing controlled ovarian hyperstimulation. Hum Reprod. 2011;26(3):671–7. 10.1093/humrep/deq361 . 21177311

[24] Homburg R, Crawford G. The role of AMH in anovulation associated with PCOS: a hypothesis. Hum Reprod. 2014;29(6):1117–21. 10.1093/humrep/deu076 . 24770999

[25] Griesinger G, Verweij PJ, Gates D, Devroey P, Gordon K, Stegmann BJ, et al Prediction of Ovarian Hyperstimulation Syndrome in Patients Treated with Corifollitropin alfa or rFSH in a GnRH Antagonist Protocol. PLoS One. 2016;11(3):e0149615 10.1371/journal.pone.0149615 ; PubMed Central PMCID: PMCPMC4780699. 26950065 PMC4780699

[26] Al-Inany HG, Youssef MA, Ayeleke RO, Brown J, Lam WS, Broekmans FJ. Gonadotrophin-releasing hormone antagonists for assisted reproductive technology. Cochrane Database Syst Rev. 2016;4:CD001750 10.1002/14651858.CD001750.pub4 . 27126581 PMC8626739

[27] Hwang JL, Chen SU, Chen HJ, Chen HF, Yang YS, Chang CH, et al Feasibility of corifollitropin alfa/GnRH antagonist protocol combined with GnRH agonist triggering and freeze-all strategy in polycystic ovary syndrome patients. J Formos Med Assoc. 2017 Epub 2017/08/24. 10.1016/j.jfma.2017.05.009 . 28830648

[28] Bosch E, Labarta E, Crespo J, Simon C, Remohi J, Jenkins J, et al Circulating progesterone levels and ongoing pregnancy rates in controlled ovarian stimulation cycles for in vitro fertilization: analysis of over 4000 cycles. Hum Reprod. 2010;25(8):2092–100. 10.1093/humrep/deq125 . 20539042

[29] Huang CC, Lien YR, Chen HF, Chen MJ, Shieh CJ, Yao YL, et al The duration of pre-ovulatory serum progesterone elevation before hCG administration affects the outcome of IVF/ICSI cycles. Hum Reprod. 2012;27(7):2036–45. Epub 2012/05/09. 10.1093/humrep/des141 . 22561057

[30] Shufaro Y, Sapir O, Oron G, Ben Haroush A, Garor R, Pinkas H, et al Progesterone-to-follicle index is better correlated with in vitro fertilization cycle outcome than blood progesterone level. Fertil Steril. 2015;103(3):669–74 e3. 10.1016/j.fertnstert.2014.11.026 . 25544249

[31] Venetis CA, Kolibianakis EM, Bosdou JK, Tarlatzis BC. Progesterone elevation and probability of pregnancy after IVF: a systematic review and meta-analysis of over 60 000 cycles. Hum Reprod Update. 2013;19(5):433–57. 10.1093/humupd/dmt014 . 23827986

[32] Chen ZJ, Shi Y, Sun Y, Zhang B, Liang X, Cao Y, et al Fresh versus Frozen Embryos for Infertility in the Polycystic Ovary Syndrome. N Engl J Med. 2016;375(6):523–33. Epub 2016/08/11. 10.1056/NEJMoa1513873 . 27509101

